# Analysis of health risk factors in the petroleum and petrochemical industry: a comprehensive study based on the analytic hierarchy process and delphi method

**DOI:** 10.3389/fpubh.2026.1911307

**Published:** 2026-10-07

**Authors:** Peiyao Shu

**Affiliations:** CNPC Research Institute of Safety and Environmental Technology, Beijing, China

**Keywords:** analytic hierarchy process, Delphi method, health risk factors, occupational health, petroleum and petrochemical industry

## Abstract

**Objective:**

This study integrates the Delphi method with the Analytic Hierarchy Process (AHP) to identify, refine, and prioritize occupational health risk factors in the petroleum and petrochemical industry. The aim is to construct a transparent sector-specific indicator hierarchy for decision support where complete and comparable plant-level data are not yet available.

**Methods:**

Twenty purposively selected experts completed two Delphi rounds. Importance was rated on a five-point scale and summarized using the mean and coefficient of variation (CV); qualitative comments were used to address conceptual overlap, measurability, and privacy concerns. Expert authority was calculated as Cr = (Ca + Cs)/2. After the indicator hierarchy was finalized, six senior experts from the original panel completed AHP pairwise comparisons. Priority weights and consistency ratios (CR) were calculated, with CR < 0.10 regarded as acceptable internal consistency.

**Results:**

All 20 experts completed both Delphi rounds. The mean expert authority coefficient was 0.813 (range 0.70–0.91). Four first-level and twelve second-level indicators were retained. Process safety risk received the highest first-level weight, followed by work environment risk, occupational behavior risk, and individual health status risk. Primary containment failure, physical hazards, and safety-system performance received the largest overall indicator weights. All AHP judgment matrices met the CR < 0.10 consistency criterion.

**Conclusion:**

The Delphi-AHP framework provides an expert-derived structure for prioritizing occupational health risks and linking them to site-level management actions. The weights are decision-support priorities rather than validated predictors of occupational illness or accidents. External validation with plant-level exposure, incident, and health-outcome data is required before broad regulatory or cross-company application.

## 1 Introduction

The petrochemical industry is critical to global energy security and economic development. However, its hazardous operational processes give rise to significant occupational health risks (1). Workers in this industry are exposed to multiple categories of risks, including chemical, physical, ergonomic, and psychosocial hazards, which impact not only workers' health and safety but also industrial productivity and sustainability.

Workers may be exposed simultaneously to hazardous chemicals, high temperature and pressure, noise, vibration, ergonomic strain, and other environmental stressors. Such exposures can affect health, quality of life, operational stability, and productivity (2–4). The management challenge is therefore not simply to list hazards, but to identify which risks warrant priority attention while preserving the distinction between occupational exposure and process-safety failure.

Given the complexity and diversity of health risk factors in the petroleum and petrochemical industry, a systematic framework integrating risk identification, screening, and prioritization is required. Existing checklist-based approaches can identify hazards but often do not quantify the relative importance of chemical, physical, behavioral, individual, and process-safety factors within one system. The central research problem is therefore how to construct a transparent and operable indicator hierarchy when complete and comparable plant-level data are unavailable.

Expert views are required because the problem crosses several professional domains. Petroleum engineers and safety managers understand process and equipment risks, occupational health professionals assess exposure and health consequences, and managers evaluate implementation feasibility. The Delphi method supports anonymous and iterative consensus, whereas the Analytic Hierarchy Process (AHP) converts the finalized indicators into quantitative priorities and tests the logical consistency of expert judgments.

Accordingly, this study combines Delphi and AHP to identify, refine, and weight health risk factors in the petroleum and petrochemical industry. The intended contribution is not a new prediction model, but a transparent bridge between multidisciplinary expert consensus and a hierarchical priority structure that integrates work environment, occupational behavior, individual health status, and process safety.

Two Delphi rounds were conducted with 20 experts, followed by AHP weighting by six senior experts selected from the original panel. Because the available research dataset did not contain standardized and comparable plant-level exposure, incident, or occupational health outcome data, the present study does not claim external predictive validation. This boundary is addressed explicitly in the implementation and future-validation sections.

The following sections review related approaches, define the objectives and scope, describe the industry risk context, explain the study methodology, present the results and discussion, and identify limitations and future research.

### 1.1 Literature review

Occupational health risk assessment has increasingly adopted multi-criteria decision-making methods to compare heterogeneous hazards. Gul and Ak developed integrated fuzzy frameworks for assessing occupational and environmental risks, demonstrating the value of combining multiple risk dimensions in complex workplaces (1, 5). Related studies have applied fuzzy AHP and other structured decision methods to improve the prioritization of occupational safety risks under uncertainty (6, 7).

A second stream of research focuses on hybrid ranking techniques. Mohandes and Zhang proposed a fuzzy-based occupational risk assessment model for construction workers (3), while La Fata et al. used VIKOR to incorporate human factors into occupational health and safety prioritization (8). Dabbagh and Yousefi combined FCM and MOORA for occupational risk analysis (9), and Abbassinia et al. (10) applied a fuzzy AHP-TOPSIS framework to emergency prioritization in a petrochemical setting. These studies show that hybrid methods can improve ranking transparency, although their indicator structures are often tailored to specific industries or decision problems.

The Delphi method has also been widely used when occupational health problems require multidisciplinary judgment. Jahanvand et al. used a fuzzy Delphi framework to determine criteria for selecting occupational risk assessment techniques (11). Ramos et al. (12) demonstrated the use of Delphi consultation in occupational safety and health analysis, and Azizi et al. (13) combined Delphi with FMEA to identify health, safety, and environmental risks in the chemical industry. These studies support the use of iterative expert consultation for indicator screening and consensus building.

Recent occupational health research emphasizes that petroleum and chemical workers face interacting chemical, physical, ergonomic, organizational, and process-related hazards (4, 14–16). Empirical exposure profiling across the petroleum supply chain and reviews of oil-and-gas worker hazards show why expert prioritization should be connected to measured workplace conditions rather than interpreted in isolation (4, 15). Yang et al. (17) developed an occupational safety and health indicator system for chemical enterprises, while a recent systematic review of OHS risk-assessment models highlights continuing methodological diversity and the importance of validation and context-sensitive application (18).

### 1.2 Comparison with alternative MCDM approaches

Method selection is consequential because different multi-criteria decision-making (MCDM) methods answer different questions. Classical AHP offers a transparent hierarchy, interpretable pairwise comparisons, and an explicit consistency test, but it assumes that the hierarchy is an adequate representation of the decision problem and can impose a substantial comparison burden. Fuzzy AHP is useful when linguistic judgments are highly uncertain; the Best-Worst Method (BWM) reduces the number of pairwise comparisons; ANP can model interdependence and feedback; Decision-Making Trial and Evaluation Laboratory (DEMATEL) is designed to map causal influence among criteria; and Preference Ranking Organization Method for Enrichment Evaluations (PROMETHEE) is particularly useful for outranking competing alternatives (6, 7, 19–22). These alternatives are summarized in Table 1.

**Table 1:** Comparison of AHP with alternative MCDM approaches.

Method	Main strength	Principal limitation	Relevance to this study
Classical AHP	Transparent hierarchy; interpretable pairwise comparisons; CR consistency test.	Many comparisons; assumes the hierarchy adequately represents relationships among criteria.	Selected because the study requires weights for a fixed goal-criterion-indicator hierarchy and auditable consistency.
Fuzzy AHP	Represents ambiguity in linguistic judgments (6, 7).	Requires fuzzy membership choices and additional computational assumptions.	Useful if uncertainty in linguistic judgments is the primary methodological concern; not required by the collected scale.
BWM	Requires fewer comparisons and provides a consistency measure (19).	Requires explicit best/worst anchors; does not by itself model causal interdependence.	A strong alternative for future sensitivity analysis and weight robustness.
ANP	Models interdependence and feedback among criteria; used in hybrid OHS models (20).	Greater cognitive and computational burden; network specification can be difficult with a small panel.	Potentially useful if future evidence demonstrates strong dependencies among the 12 indicators.
DEMATEL	Maps directional influence and causal relationships among risk factors (22).	Produces an influence structure rather than a simple hierarchical weighting scheme.	Useful for future analysis of how process, behavior, and exposure risks influence one another.
PROMETHEE	Supports outranking of alternatives and can be integrated with fuzzy information (21).	Requires alternatives and preference functions/thresholds.	Less aligned with the current aim, which is indicator weighting rather than ranking competing plants or interventions.

The comparison clarifies methodological fit; it does not imply that one MCDM method is universally superior.

The present study retained Delphi-AHP because the immediate task is to screen a multidisciplinary indicator set and derive transparent weights for a fixed three-level hierarchy, rather than to rank competing plants or interventions. Delphi provides iterative indicator refinement, while AHP transforms the finalized hierarchy into priorities that can be audited through consistency ratios. This choice favors interpretability and implementability under limited plant-level data; it does not imply that AHP is universally superior to fuzzy, network, causal, or outranking methods. Recent systematic evidence also emphasizes that OHS risk-assessment models should ultimately be linked to empirical exposure and outcome information, a requirement incorporated into the external-validation protocol proposed below (18).

Despite these advances, four gaps remain. First, many models assess occupational exposure or process safety separately and do not integrate both within one hierarchy. Second, the transfer from Delphi screening to AHP weighting is often insufficiently documented. Third, method selection is not always compared explicitly with alternative MCDM approaches. Fourth, expert-derived weights are frequently reported without a practical implementation pathway or sufficient discussion of external validation and panel dependence. The present study addresses these gaps through a documented sequential Delphi-AHP procedure, an explicit methodological comparison, a site-level implementation framework, and a bounded interpretation of the resulting weights.

### 1.3 Objectives and scope of work

The objectives are to: (1) establish a multidimensional health risk indicator system; (2) screen and refine the indicators through two Delphi rounds; (3) calculate indicator weights through AHP; and (4) identify priority areas for occupational health management. The study covers work environment, occupational behavior, individual health status, and process safety risks. It ranks risk factors rather than intervention solutions and does not estimate individual disease probability.

### 1.4 Health risk problems in the petroleum and petrochemical industry

The petroleum and petrochemical industry includes exploration and production, transportation and storage, refining and chemical processing, and product distribution (10). Occupational hazards differ across these stages but are interconnected. Upstream activities may involve pressure, remote work, hydrogen sulfide, and fatigue; midstream activities involve transport and storage risks; and downstream operations involve heat, noise, vibration, volatile chemicals, complex equipment, and process deviations.

A closed-loop risk-management process can therefore be expressed as follows: operational activities, hazard identification, expert screening, AHP prioritization, control planning, monitoring and feedback, reassessment. This process links the types of hazards occurring at different operational stages with the selection and periodic revision of control priorities.

This study focuses on occupational health and process-related risks that can affect workers during petroleum and petrochemical operations, including toxic exposure, physical hazards, unsafe behavior, ergonomic strain, individual susceptibility, equipment failure, safety-system performance, and abnormal process parameters.

## 2 Methodology

### 2.1 Delphi method

The Delphi method is a structured process for developing expert judgment through repeated, anonymous consultation and controlled feedback. It is especially appropriate when a decision problem spans several professional domains and direct empirical evidence is incomplete or heterogeneous (12, 23, 24).

In this study, Delphi consultation was used to refine the content and boundaries of the indicator system before any AHP weighting was performed. This sequencing reduces the risk that numerical weighting is applied to indicators that are poorly defined, duplicative, difficult to measure, or inappropriate for routine occupational health management.

The initial indicator pool was developed from the literature, international occupational health frameworks, and semi-structured interviews. The same 20 experts completed two rounds of five-point importance ratings and qualitative review. No single automatic numerical cutoff determined deletion in Round 1; mean importance, dispersion, conceptual overlap, measurability, privacy concerns, and written expert explanations were considered jointly.

### 2.2 Analytic hierarchy process

AHP was applied only after the Delphi indicator system had been finalized. The procedure was: (1) define the goal, criterion, and indicator levels; (2) obtain pairwise comparisons using Saaty's scale; (3) aggregate the expert comparison values; (4) calculate the priority vector and local/global weights; and (5) test each matrix for consistency. A matrix was accepted when the consistency ratio (CR) was below 0.10.

The finalized Delphi hierarchy was transferred unchanged to the AHP stage. Six senior experts from the original 20-member panel completed the pairwise comparisons because they had participated in both Delphi rounds and collectively covered petroleum engineering, safety/health management, and research roles. Their comparison values were aggregated using the arithmetic mean, consistent with the original data-collection and calculation procedure used in this study.

### 2.3 Data and statistical analysis

The dataset contained expert demographics, judgment-basis and familiarity scores, five-point importance ratings, coefficient of variation (CV) values, qualitative revision comments, and AHP pairwise-comparison values. For Delphi summaries, the arithmetic mean described perceived importance and CV = SD/mean described relative dispersion. Expert authority was calculated as Cr = (Ca + Cs)/2, where Ca denotes the coefficient for judgment basis and Cs denotes familiarity. The reported statistics are descriptive only and do not support population-level inference; therefore, the chi-square test result was not presented because the original data lacked a reproducible test statistic and corresponding degrees of freedom. Excel was used for data organization, SPSS for descriptive summaries, and Yaahp for AHP matrices, weights, eigenvalues, and consistency ratios.

### 2.4 Reproducibility and decision rules

To enable replication, the analytical sequence is fixed as follows: generate the preliminary indicators from the documented sources; administer the same five-point importance scale to the 20-member panel; review Round-1 mean scores, CV values, and qualitative reasons together; revise the indicator wording and remove indicators only when the quantitative pattern is supported by a substantive rationale; re-rate the revised system in Round 2; freeze the retained hierarchy; obtain pairwise comparisons from the six AHP experts; aggregate pairwise values; calculate eigenvector-based weights; and accept only matrices with CR < 0.10. The pairwise-comparison scale and random consistency indices are reported in Tables 2, 3, and the resulting matrices are reported in Tables 4, 5.

**Table 2:** Saaty pairwise-comparison scale.

Scale	Meaning
1	The two factors are of equal importance.
3	Compared with the latter, the former factor is slightly more important.
5	Compared with the latter, the former factor is clearly more important.
7	Compared with the latter, the former factor is strongly more important.
9	Compared with the latter, the former factor is extremely more important.
2, 4, 6, 8	Intermediate values between two adjacent judgments.
Reciprocal of the above values	When the comparison order of the two factors is reversed, the corresponding value is the reciprocal of the original judgment.

**Table 3:** Average random consistency index (RI).

n	1	2	3	4	5	6	7	8	9	10	11	12	13	14
R.I.	0	0	0.52	0.89	1.12	1.24	1.36	1.41	1.46	1.49	1.52	1.54	1.56	1.58

**Table 4:** Pairwise-comparison matrix of first-level indicators and corresponding weights.

A-B	B1	B2	B3	B4	Weights
B1	1	2	3	1/2	0.2926
B2	1/2	1	2	1/2	0.1849
B3	1/3	1/2	1	1/3	0.1070
B4	2	2	3	1	0.4155
λ_max_= 4.0710	C.I.=0.0237				C.R. = 0.0266

**Table 5:** Pairwise-comparison matrices for the indicator level.

B1–C	C1	C2	C3	Weights
C1	1	3	4	0.6337
C2	1/3	1	1	0.1919
C3	1/4	1	1	0.1744
λ_max_= 3.0092	C.I.=0.0046			C.R.=0.0088
B2**–**C	C4	C5	C6	Weights
C4	1	2	1/2	0.2970
C5	1/2	1	1/3	0.1634
C6	2	3	1	0.5396
λ_max_= 3.0092	C.I.=0.0046			C.R.=0.0088
B3**–**C	C7	C8	C9	Weights
C7	1	1	2	0.4000
C8	1	1	2	0.4000
C9	1/2	1/2	1	0.2000
λ_max_= 3.0000	C.I.=0.0000			C.R.=0.0000
B4**–**C	C10	C11	C12	Weights
C10	1	2	5	0.5816
C11	1/2	1	3	0.3090
C12	1/5	1/3	1	0.1095
λ_max_= 3.0037	C.I.=0.0018			C.R.=0.0036

A formal Kendall concordance analysis could not be performed due to the lack of rank-level data; therefore, reduced CVs are interpreted as greater convergence rather than statistical proof of consensus. Future studies should retain complete round-level raw data and prespecify a concordance analysis when inferential assessment of expert agreement is intended (23, 24).

### 2.5 Expert panel overview

#### 2.5.1 Study sample

Twenty experts were purposively selected to provide informed perspectives on petroleum operations, safety, occupational health, and risk management. Table 6 shows that all experts had 12–25 years of work experience; 13 were men and 7 were women; 11 reported onshore experience, 5 offshore experience, and 4 both. The panel was nevertheless engineering-heavy: 16 of 20 experts (80%) were categorized as petroleum engineering, while four represented occupational safety/health or occupational health risk assessment. The panel was designed for focused expert consensus rather than statistical representativeness.

**Table 6:** Demographic characteristics of experts.

ID	Sex	Age	Work exp. (years)	Country	Operational setting	Education	Position	Specialty
N1	Male	41	15	China	Onshore	Master's	Senior engineer	Petroleum engineering
N2	Male	42	16	China	Both	Ph.D.	Professor	Petroleum engineering
N3	Male	40	15	China	Onshore	Ph.D.	Senior engineer	Petroleum engineering
N4	Female	39	16	China	Onshore	Master's	Manager	Petroleum engineering
N5	Male	45	17	China	Offshore	Bachelor's	Senior professional technician	Petroleum engineering
N6	Female	38	14	China	Onshore	Ph.D.	Senior researcher	Petroleum engineering
N7	Male	44	18	China	Both	Master's	Senior engineer	Petroleum engineering
N8	Male	43	19	China	Offshore	Bachelor's	Operations manager	Petroleum engineering
N9	Female	37	13	China	Onshore	Ph.D.	Project lead	Petroleum engineering
N10	Male	46	20	China	Both	Master's	Senior engineer	Petroleum engineering
N11	Male	40	15	China	Onshore	Master's	Senior safety officer	Occupational safety & health
N12	Female	42	17	China	Onshore	Ph.D.	Professor	Occupational health
N13	Male	48	22	China	Offshore	Bachelor's	Senior drilling engineer	Petroleum engineering
N14	Female	36	12	China	Onshore	Master's	Safety manager	Occupational safety & health
N15	Male	41	16	China	Onshore	Ph.D.	Research scientist	Occupational health risk assessment
N16	Male	47	21	China	Offshore	Bachelor's	Field manager	Petroleum engineering
N17	Female	40	15	China	Onshore	Ph.D.	Senior engineer	Petroleum engineering
N18	Male	39	14	China	Onshore	Master's	Project engineer	Petroleum engineering
N19	Female	44	19	China	Both	Ph.D.	Head of operations	Petroleum engineering
N20	Male	50	25	China	Offshore	Bachelor's	Senior engineer	Petroleum engineering

#### 2.5.2 Selection principles

Expert selection emphasized direct knowledge of petroleum and petrochemical operations, disciplinary relevance to health or process risk, familiarity with current operating conditions, and the ability to judge whether proposed indicators were both scientifically meaningful and operationally usable. Experts were recruited purposively from petroleum-industry and related professional settings to provide operational, safety, health, management, environment, and research perspectives. Recruitment was not random; the purpose was to obtain informed judgments for indicator development rather than to estimate population parameters.

Senior operational or managerial experience and willingness to complete repeated consultation with reasoned feedback were also considered because the framework is intended to inform resource allocation and risk-control priorities. Purposive selection improves contextual expertise but does not eliminate sampling limitations. The final panel included different genders, ages, educational backgrounds, and operational settings, but all 20 experts were based in China and affiliated with CNPC or related institutions, and petroleum-engineering specialists predominated. The study therefore treats their judgments as context-specific expert evidence rather than statistically representative of the global petroleum and petrochemical workforce or all enterprise types.

#### 2.5.3 Panel characteristics and representativeness

The panel varied in age, education, position, and operational setting, but its institutional and disciplinary concentration is an important boundary condition. Tables 6, 7 provide the individual demographic and authority information needed to evaluate who contributed to the consensus process. Generalization to other companies or countries should be tested with independent panels.

**Table 7:** Expert authority level.

Expert	Judgment basis (Ca)	Familiarity (Cs)	Authority coefficient (Cr)
N1	1	0.8	0.9
N2	0.9	0.8	0.85
N3	0.8	0.8	0.8
N4	0.85	0.6	0.73
N5	0.85	0.8	0.83
N6	0.9	0.75	0.88
N7	0.95	0.9	0.9
N8	0.8	0.85	0.78
N9	0.7	0.7	0.75
N10	0.85	0.65	0.77
N11	0.88	0.82	0.82
N12	0.92	0.88	0.86
N13	0.78	0.9	0.76
N14	0.86	0.72	0.79
N15	0.9	0.74	0.84
N16	0.95	0.87	0.91
N17	0.81	0.65	0.7
N18	0.77	0.77	0.74
N19	0.83	0.8	0.8
N20	0.9	0.78	0.85

#### 2.5.4 Theoretical basis for indicator construction

The preliminary indicator pool was informed by international occupational health and safety frameworks and sector-specific literature. ILO-oriented risk management emphasizes systematic identification of physical, chemical, biological, and psychosocial hazards and the selection of preventive controls, while international occupational safety approaches also emphasize hazard identification, exposure assessment, risk characterization, and iterative management (14). The WHO healthy-workplace perspective further supports assessment at both individual and organizational levels. In petroleum and petrochemical settings, this rationale requires attention not only to exposure hazards but also to behavior, individual susceptibility, equipment integrity, and process-control conditions (4).

Semi-structured interviews with safety managers, occupational health physicians, front-line operators, and technical experts were used to identify sector-specific concerns not fully captured by generic frameworks. Interviewees emphasized the connection between process-safety failures and acute exposure, the role of baseline health and lifestyle in susceptibility, fatigue and ergonomic strain associated with work organization, and the importance of equipment condition and process-parameter deviations. These themes were used to expand and clarify the preliminary indicator pool rather than to generate quantitative weights.

The preliminary system therefore covered work environment hazards, occupational behavior risks, individual health status, and process-safety risks. Exploratory indicators were retained in Round 1 so that experts could judge their relevance rather than having them removed *a priori*. Table 8 reports the preliminary indicator system, and Figure 1 presents the final hierarchical structure used after Delphi refinement.

**Table 8:** Preliminary health risk assessment indicator system for the petrochemical industry.

First-level indicator	Second-level indicator	Description of core indicator content
Work environment risk	Physical hazards	Acute and chronic health impacts of physical factors such as noise, high temperature, vibration, and radiation in the working environment.
	Chemical exposure	Occupational exposure risks to toxic and hazardous chemicals, including benzene compounds, hydrogen sulfide, and volatile organic compounds (VOCs), through inhalation, dermal contact, and other pathways.
	Biological exposure	Health threats posed by harmful biological agents, such as molds and bloodborne pathogens, in specific operational scenarios (e.g., tank cleaning and wastewater treatment).
	Environmental psychological load	Potential stress and anxiety risks to employees' mental health caused by monotonous working environments and feelings of isolation.
Occupational behavior risk	Ergonomic hazards	Risks of musculoskeletal disorders resulting from repetitive tasks, improper working postures, and manual handling of heavy loads.
	Working time and rhythm risk	Disruptions of circadian rhythms and cumulative fatigue caused by prolonged working hours, shift work systems, and insufficient rest.
	Unsafe behavioral risk	Health damage arising from human factors such as failure to use personal protective equipment (PPE), violations of operating procedures, and improper emergency responses.
	Lack of social support	Reduced psychological support due to social isolation caused by shift work and inadequate communication within work teams.
Individual health status risk	Baseline health risk	Increased susceptibility to occupational hazards due to employees' chronic diseases (e.g., cardiovascular and respiratory conditions) and age-related physiological decline.
	Lifestyle-related risk	Additional negative impacts on occupational health associated with smoking, alcohol consumption, unhealthy diets, and lack of physical activity.
	Individual susceptibility factors	Differences in sensitivity to specific toxic substances attributable to biological characteristics such as genetic metabolism, age, and gender.
	Family history of genetic diseases	The role of familial genetic disease history in identifying occupational contraindications and predicting potential health risks.
Process safety risk	Primary containment failure risk	Risks of material contact and secondary toxic gas dispersion caused by leakage or failure of equipment such as storage tanks, pipelines, and reactors.
	Safety system performance risk	Increased accumulation and exposure to hazardous factors due to malfunction or failure of protective systems such as ventilation, alarm systems, and emergency showers.
	Process parameter control risk	Deterioration of the working environment and abnormal emissions resulting from deviations in key process parameters, including temperature, pressure, and concentration.

**Figure 1:**
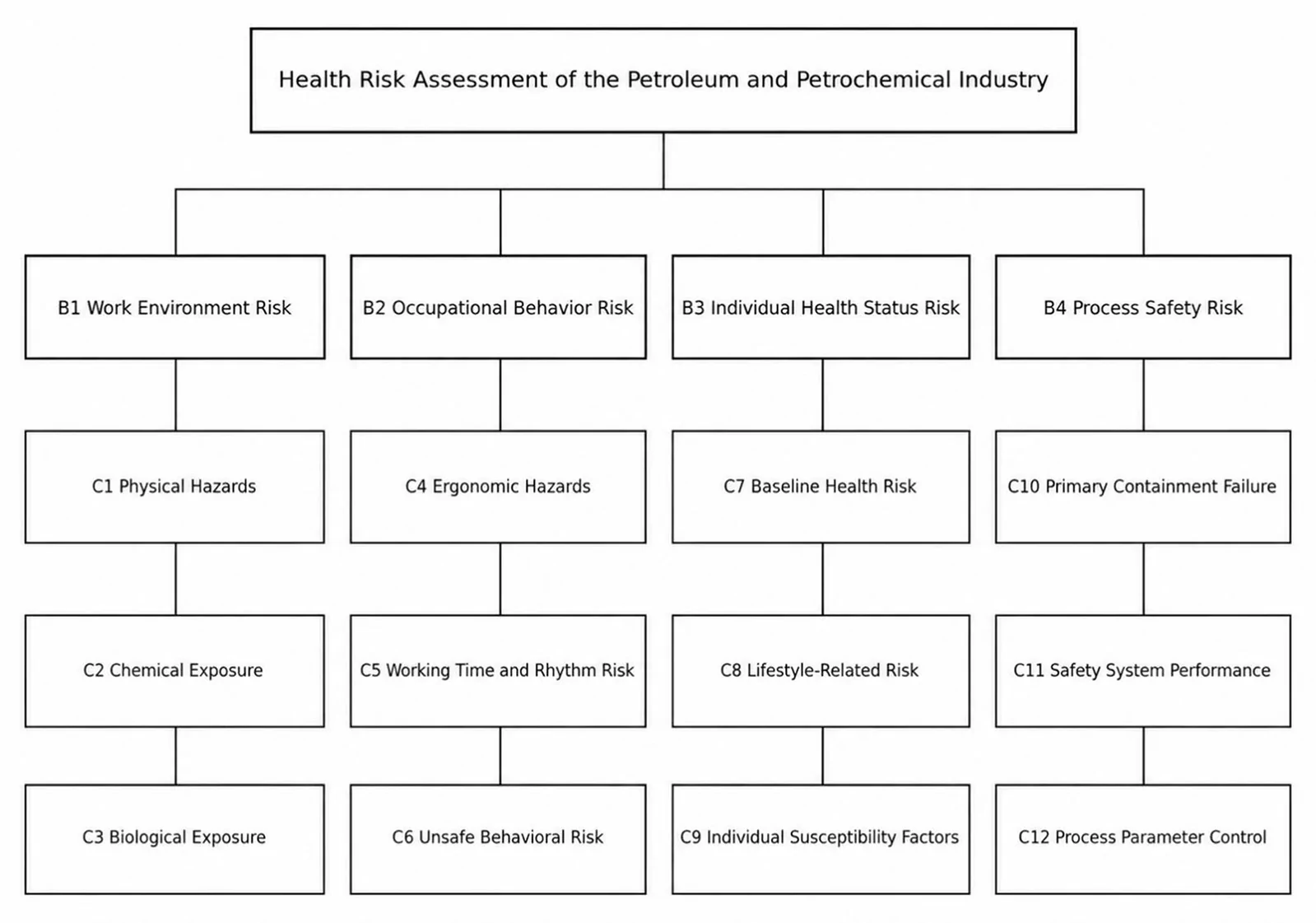
Final Delphi-AHP hierarchy of health risk factors in the petroleum and petrochemical industry.

## 3 Results

### 3.1 Delphi consultation results

#### 3.1.1 First-round expert consultation

- A. *Questionnaire Design and Distribution*

Based on the initially constructed index system, the first round of expert questionnaires was designed. The questionnaires included all the initially proposed indicators and required experts to rate the importance of each indicator on a 5-point scale (1—not important, 2—not too important, 3—moderately important, 4—important, 5—very important), and also requested experts to propose suggestions and reasons for modifying the indicators.

- B. *Recipient Results and Analysis*

All 20 experts returned valid Round-1 questionnaires (100% response). Across the preliminary indicators, the average importance score was 4.05, and 82.3% of indicators had CV < 0.25 (Table 9). These statistics suggested generally high importance ratings with some residual dispersion. Three exploratory indicators attracted low mean scores and substantive concerns regarding conceptual fit, overlap, measurability, or privacy, and were therefore reviewed for deletion.

**Table 9:** Statistical results of the first-round Delphi consultation.

First-level indicator	Second-level indicator	Mean importance score (M)	Coefficient of variation (CV)
Work environment risk	Physical hazards	4.6	0.11
	Chemical exposure	4.7	0.1
	Biological exposure	4.2	0.16
	Environmental psychological load	3.1	0.27
Occupational behavior risk	Ergonomic hazards	4.4	0.13
	Working time and rhythm risk	4.5	0.13
	Unsafe behavioral risk	4.6	0.13
	Lack of social support	2.9	0.33
Individual health status risk	Baseline health risk	4.3	0.13
	Lifestyle-related risk	4.1	0.19
	Individual susceptibility factors	4	0.18
	Family history of genetic diseases	2.6	0.32
Process safety risk	Primary containment failure risk	4.5	0.11
	Safety system performance risk	4.4	0.13
	Process parameter control risk	4.3	0.15

Environmental psychological load received a mean score of 3.1. Experts considered its direct link to occupational health and safety relatively weak and noted that psychological stress was more appropriately represented through organizational or management factors. The indicator was therefore removed.

Lack of social support received a mean score of 2.9. Experts noted that the influence of shift work on social interaction overlapped with working-time and rhythm risk, so a separate indicator would duplicate content. It was therefore removed.

Family history of genetic disease received the lowest mean score (2.6). Experts raised concerns about privacy, implementation feasibility, and limited relevance to current job-specific control. It was therefore removed from the final occupational health risk indicator set.

#### 3.1.2 Second-round expert consultation

##### 3.1.2.1 Questionnaire adjustment and design

Based on Round-1 results, Environmental Psychological Load, Lack of Social Support, and Family History of Genetic Disease were removed because of low importance ratings and the substantive concerns described above. Other indicators were retained, while labels and descriptions were simplified for clarity; for example, the wording for the primary containment/LOPC indicator was revised to emphasize equipment failure and leakage. Round 2 asked experts to re-rate the revised indicators and comment on the proposed weighting principles.

##### 3.1.2.2 Recovery results and analysis

The revised questionnaire was sent to the same 20 experts, and all 20 returned valid responses (100% response). Every retained indicator had a mean importance score above 4.0 and CV < 0.20. Relative to Round 1, this pattern indicates lower dispersion around high importance ratings and supported finalization of the indicator set. It should not be interpreted as evidence of external validity or predictive accuracy.

Based on the Round-2 results, all retained indicators had high importance scores and low relative dispersion (Table 10). The finalized health risk assessment indicator system for the petroleum and petrochemical industry is reported in Table 11.

**Table 10:** Statistical results of the second-round Delphi consultation.

First-level indicator	Second-level indicator	Mean importance score (m)	Coefficient of variation (CV)
Work environment risk	Physical hazards	4.6	0.11
	Chemical exposure	4.8	0.12
	Biological exposure	4.3	0.11
Occupational behavior risk	Ergonomic hazards	4.5	0.11
	Working time and rhythm risk	4.6	0.11
	Unsafe behavioral risk	4.7	0.11
Individual health status risk	Baseline health risk	4.4	0.13
	Lifestyle-related risk	4.2	0.15
	Individual susceptibility factors	4.2	0.14
Process safety risk	Primary containment failure risk	4.5	0.11
	Safety system performance risk	4.5	0.11
	Process parameter control risk	4.4	0.13

M represents the mean importance score, and CV denotes the coefficient of variation.

**Table 11:** Final health risk assessment indicator system for the petroleum and petrochemical industry.

Target level	Criteria level	Indicator level
Health risk assessment of the petrochemical industry (A)	Work environment risk (b1)	Physical hazards (c1)
		Chemical exposure (c2)
		Biological exposure (c3)
	Occupational behavior risk (b2)	Ergonomic hazards (c4)
		Working time and rhythm risk (c5)
		Unsafe behavioral risk (c6)
	Individual health status risk (b3)	Baseline health risk (c7)
		Lifestyle-related risk (c8)
		Individual susceptibility factors (c9)
	Process safety risk (b4)	Primary containment failure risk (c10)
		Safety system performance risk (c11)
		Process parameter control risk (c12)

### 3.2 Calculation of indicator weights

#### 3.2.1 Methods for determining indicator weights

The weight of an evaluation index refers to the degree of influence that each index has on the overall goal within the comprehensive evaluation index system. A higher weight indicates a greater influence of the index on the overall goal; a lower weight indicates a smaller influence. Currently, the methods for determining weights can be mainly divided into two categories:

**1) Subjective weighting methods**

Subjective weighting methods include Delphi-based scoring, AHP, BWM, and related expert-judgment approaches. Their main advantage is the ability to incorporate domain knowledge when direct measurements are heterogeneous or unavailable; their main limitation is dependence on panel composition and judgment structure (18, 19).

**2) Objective weighting methods**

Objective weighting approaches, including entropy-based methods and some data-driven dimension-reduction procedures, derive weights primarily from observed data structure. They can reduce direct dependence on expert preference, but they require sufficiently complete and comparable quantitative data and do not by themselves ensure that statistically variable indicators are clinically or operationally important.

For the present study, comparable plant-level measurements were not available across the physical, chemical, biological, behavioral, individual-health, and process-safety domains. A purely data-driven weighting method would therefore create an appearance of objectivity without a common measurement base. AHP was selected because it can combine expert knowledge with an explicit hierarchy and consistency test. This choice is appropriate for constructing provisional decision-support priorities, but the resulting weights remain expert-derived and require empirical validation.

#### 3.2.2 Weight determination using AHP

##### 3.2.2.1 Main steps of AHP

Firstly, construct the hierarchical analysis model by dividing the indicator system into the goal layer, criterion layer and indicator layer.

Second, construct the judgment matrix. For each level, compare indicators in pairs using Saaty's two-way comparison scale reported in Table 2.

Finally, a consistency test was conducted. The purpose of the consistency test is to examine whether the judgment matrix is reasonable and free from logical inconsistencies. If logical inconsistencies are detected, the judgment matrix must be revised and adjusted until the consistency requirement is satisfied:


$$\begin{array}{l} C.I.=\frac{\lambda_{\max}-n}{n-1} \end{array}$$ (1)

Here,λ_max_ represents the maximum eigenvalue of the judgment matrix, and *n* denotes the dimension of the judgment matrix. The larger the value of the consistency index C.I., the greater the degree to which the judgment matrix deviates from complete consistency. The smaller the C.I., the closer the judgment matrix is to complete consistency.

For multi-level judgment matrices, the average random consistency index (RI) is introduced. Table 3 reports the RI values for positive reciprocal matrices of orders 1 to 15.

The random consistency ratio of the judgment matrix is called C.R.:


$$\begin{array}{l} C.R.=\frac{C.I.}{R.I.} \end{array}$$ (2)

When C.R. < 0.10, it is considered that the judgment matrix has acceptable consistency. When C.R. ≥ 0.10, the judgment matrix needs to be adjusted and corrected to achieve acceptable consistency.

##### 3.2.2.2 Calculation of indicator weights

Six senior experts from the original Delphi panel completed the AHP pairwise comparisons. The recorded pairwise values were aggregated by arithmetic mean to construct each group judgment matrix A = (aij). For each matrix, the priority vector was obtained using the principal-eigenvector method: the right eigenvector w satisfying Aw = λmaxw was calculated and normalized so that the weights summed to 1. Local weights were derived at each hierarchical level, and each second-level indicator's overall weight was calculated by multiplying its local weight by the weight of its corresponding first-level criterion. This procedure follows the standard AHP weighting logic described by Saaty (25) and Sutadian et al. (26). The pairwise-comparison scale, group matrices, and consistency results are reported in Tables 2–5, allowing the reported weights to be reproduced from the documented matrices.

For the first-level and indicator-level judgment matrices (Tables 4, 5), all consistency ratios were below 0.10 (A-B: CR = 0.0266; B1-C: 0.0088; B2-C: 0.0088; B3-C: 0.0000; B4-C: 0.0036), indicating acceptable internal consistency of the pairwise judgments. These consistency results do not constitute external validation of the risk model.

The resulting weights for the petroleum and petrochemical health risk assessment system are reported in Table 12.

**Table 12:** Weighting results of the health risk assessment indicator system.

Target level	Criteria level	Indicator level	Within-group weight	Overall weight
Health risk assessment of the petrochemical industry (A)	Work environment risk (b1)		0.2926	
		Physical hazards (c1)	0.6337	0.1854
		Chemical exposure (c2)	0.1919	0.0561
		Biological exposure (c3)	0.1744	0.051
	Occupational behavior risk (b2)		0.1849	
		Ergonomic hazards (c4)	0.297	0.0549
		Working time and rhythm risk (c5)	0.1634	0.0302
		Unsafe behavioral risk (c6)	0.5396	0.0998
	Individual health status risk (b3)		0.107	
		Baseline health risk (c7)	0.4	0.0428
		Lifestyle-related risk (c8)	0.4	0.0428
		Individual susceptibility factors (c9)	0.2	0.0214
	Process safety risk (b4)		0.4155	
		Primary containment failure risk (c10)	0.5816	0.2417
		Safety system performance risk (c11)	0.309	0.1284
		Process parameter control risk (c12)	0.1095	0.0455

## 4 Discussion

The AHP results show clear differences among the indicator weights. Process safety risk ranks first, followed by work environment risk, occupational behavior risk, and individual health status risk. These findings are interpreted below in relation to occupational health management and previous research:

### 4.1 First-level indicator priorities

Within this expert panel, the high priority assigned to process safety reflects the potential for loss of containment, equipment failure, safety-system malfunction, and process deviations to generate acute, high-consequence exposure events. Work environment risk ranks second because noise, heat, vibration, and hazardous substances can produce persistent exposure across routine operations. The ordering should be interpreted as a management-priority structure derived from this panel, not as a universal ordering of toxicological severity.

### 4.2 Secondary-indicator priorities

Within the work environment domain, physical hazards received a higher weight than chemical and biological exposures. This result may reflect the experts' perception that noise, heat, and vibration are widespread across many positions. It should not be interpreted as evidence that toxic chemicals such as benzene, hydrogen sulfide, or volatile organic compounds are less harmful. The ranking may be influenced by the operational experience and institutional composition of the panel and should be compared with plant-specific exposure data.

Within occupational behavior risk, unsafe behavioral risk received a higher weight than ergonomic hazards and working-time/rhythm risk. The result indicates that the panel prioritized failures such as incorrect protective-equipment use and non-compliance with operating procedures. However, this does not imply that ergonomic strain or fatigue is negligible; these factors may become more prominent in sites or occupations with different task structures.

Within process safety, primary containment failure received the largest weight, followed by safety-system performance and process-parameter control. This pattern places equipment integrity and leakage prevention at the center of acute risk management, while also recognizing the protective role of alarms, interlocks, emergency systems, and stable operating parameters.

Within individual health status, baseline health and lifestyle-related risk received equal weights, while individual susceptibility factors received a smaller weight. These priorities support confidential health surveillance and fitness-for-work management as complements to, rather than substitutes for, exposure prevention and engineering control.

Across the two Delphi rounds, the retained indicators showed high importance ratings and lower relative dispersion in Round 2. This pattern is consistent with convergence of expert views, but the study does not claim formal statistical consensus beyond the reported descriptive evidence. AHP then translated the finalized hierarchy into internally consistent relative priorities, allowing the contribution of each criterion and indicator to be compared on a common scale.

The priority structure is broadly compatible with literature describing chemical exposure, physical hazards, ergonomic strain, unsafe behavior, and process-related risks in petroleum and chemical operations (4, 14–18). Nevertheless, compatibility with prior literature is not a substitute for external validation. The current weights were not derived from measured benzene, hydrogen sulfide, noise, heat, incident, or health-outcome data, and should therefore be treated as hypotheses about relative management priority that require site-level testing.

For practitioners, the ranking indicates that equipment integrity, leakage prevention, safety-system reliability, exposure control, safe-work behavior, ergonomics, fatigue management, and confidential employee health management should be considered within one coordinated system. The practical question is how to convert the global weights into site-specific action; Figure 2 and Table 13 provide an implementation procedure and an illustrative calculation.

**Figure 2:**
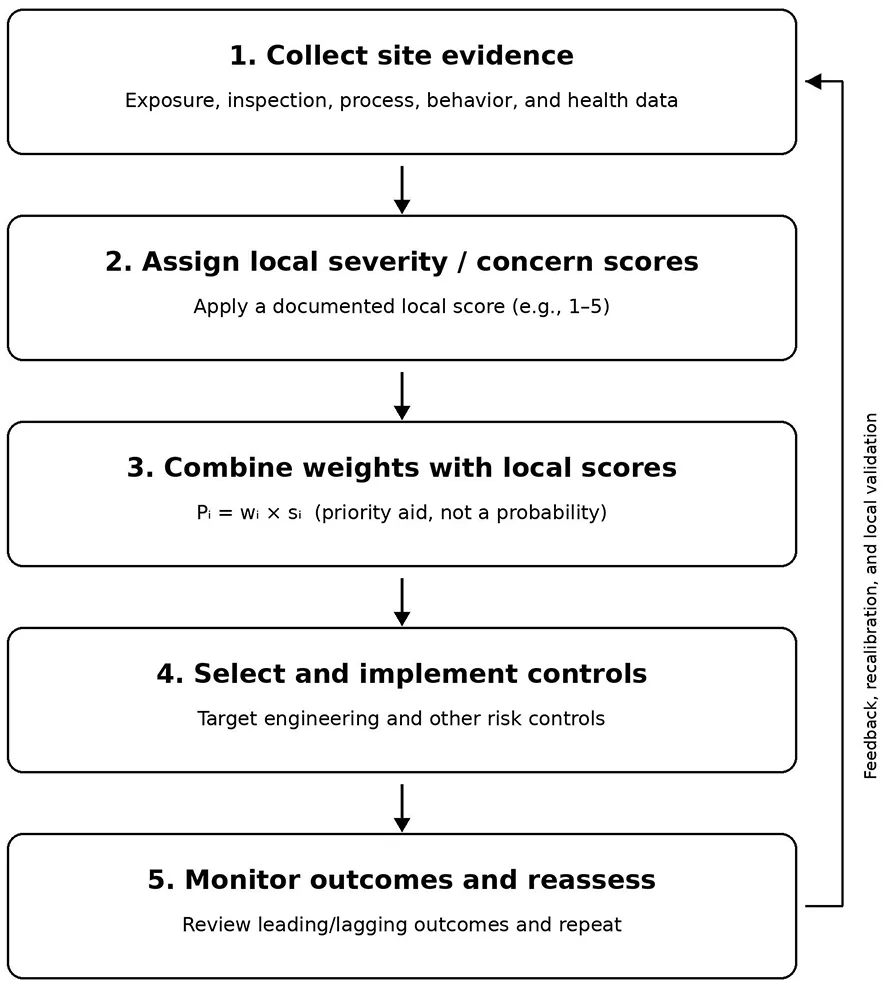
Practical implementation and external-validation cycle for enterprise use of the expert-derived weights.

**Table 13:** Illustrative site-level use of the overall indicator weights.

Indicator	Overall weight (w_i_)	Illustrative site score (s_i_)	Priority index (w_i_ × s_i_)	Illustrative management response
C10 Primary containment failure	0.2417	5	1.2085	Prioritize containment integrity inspection, leak prevention, and maintenance.
C1 Physical hazards	0.1854	4	0.7416	Strengthen noise, heat, and vibration monitoring and controls.
C11 Safety–system performance	0.1284	3	0.3852	Test alarms, interlocks, emergency shutdown, and related protective systems.

Scores are hypothetical and are provided only to demonstrate implementation. They are not plant data and do not constitute external validation.

### 4.3 Practical implementation framework

Enterprises can use the weights in a five-step cycle. First, collect site evidence for each of the 12 indicators using exposure monitoring, inspection findings, process-safety data, work-practice observations, and confidential occupational-health information. Second, assign a site-specific condition score (for example, 1 = low concern to 5 = very high concern) using a documented scoring rubric. Third, calculate a priority index P_i_ = w_i_ × s_i_, where w_i_ is the overall AHP weight and s_i_ is the local score. Fourth, target higher-priority indicators with controls consistent with the hierarchy of controls. Fifth, monitor leading and lagging indicators and repeat the assessment periodically or after major process, equipment, or organizational change. The multiplication is a management prioritization aid, not a validated probability equation.

Table 13 provides a hypothetical illustration. If a plant assigned scores of 5 to primary containment failure (C10), 4 to physical hazards (C1), and 3 to safety-system performance (C11), the corresponding priority indices would be 1.2085, 0.7416, and 0.3852. Under that scenario, containment integrity would receive the first management response. The example is intentionally illustrative and does not constitute empirical validation of the weights.

### 4.4 External validation protocol

External validation should proceed prospectively across multiple enterprises. For each site, the weighted priority profile should be compared with independent evidence such as measured exposure concentrations, noise/heat/vibration measurements, loss-of-containment and near-miss records, safety-system demands or failures, occupational health surveillance, and incident outcomes. Validation should examine rank correspondence, temporal stability, discrimination between higher- and lower-burden sites or units, and sensitivity of priorities to plausible changes in expert weights.

A stronger design would use geographically and institutionally independent expert panels to recalculate the weights, then test whether the ranking remains stable across organizations and regulatory contexts. Where measured data reveal systematic disagreement with the expert-derived order, the hierarchy or weights should be recalibrated rather than treated as fixed. Figure 2 summarizes this implementation-validation loop.

## 5 Conclusion

This study establishes a Delphi-AHP occupational health risk assessment system containing four first-level and twelve second-level indicators for the petroleum and petrochemical industry. Its principal contribution is a transparent sequence linking indicator development, expert refinement, hierarchical weighting, consistency testing, and a practical site-level prioritization cycle. Process safety received the highest first-level priority, and primary containment failure received the largest overall indicator weight. These values should be interpreted as expert-derived decision-support priorities rather than validated estimates of disease or accident probability. Before broad application, the framework should be tested with independent panels and plant-level exposure, incident, and health-outcome data.

## 6 Limitations and future research

The study has six main limitations. First, the Delphi panel included only 20 experts, which is appropriate for a focused expert-consensus design but not for population inference. Second, all experts were based in China and were affiliated with CNPC or related institutions, limiting institutional and geographic transferability. Third, the panel was engineering-heavy: 16 of 20 experts were categorized as petroleum engineering. Fourth, only six experts completed the AHP comparisons. Fifth, no plant-level exposure, near-miss, incident, or occupational health outcome data were available for external validation. Sixth, the retained dataset did not permit a reproducible formal concordance test or sensitivity analysis. Future research should use multi-company and multinational panels, retain complete round-level data, compare AHP weights with BWM/fuzzy/network alternatives where appropriate, conduct sensitivity analysis, and prospectively validate the framework in operating plants.

## Data Availability

The original contributions presented in the study are included in the article/supplementary material, further inquiries can be directed to the corresponding author.
